# Going to the South

**Published:** 1854-02

**Authors:** 


### GOING TO THE SOUTH.

The colder the out-door air is, the purer it must be, and, therefore, more healthful and invigorating; not only is it more healthful in consequence of its freedom from impurities, but also from the concentration of its life-giving property, because air is condensed by cold; it is packed, as it were, more solid; so that, even supposing two cubic inches of air equally pure, one at the equator, the other at the poles, the one at the poles has a much larger amount of oxygen, the great life-giver and purifier of the blood.

If, therefore, a man is really consumptive, a warmer climate will inevitably hasten his death; and it is wonderful, that it continues to be the stereotyped advice given by northern medical and non-medical men, without the slightest consideration of the ability of the patient to meet the expenses of such a journey; and more, without any opportunity of personally observing, on the spot, whether such advice is for life or death.

